# Editorial Board Acknowledgment

**DOI:** 10.1097/CCE.0000000000001377

**Published:** 2026-03-09

The Editors of *Critical Care Explorations* thank the following physicians and healthcare professionals for contributing their time and expertise to the review of the manuscripts submitted to the journal from 2025. Peer review is one of the most important cornerstones assuring the publication of the highest quality science and medical research. The Editors and the staff of the journal sincerely appreciate the enormous amount of time and effort expended in the past year by these, our outstanding reviewers.

Kenneth Abbott, MD

Peter Abdelmalik, MD, PhD

Alaa Abu Sayf, MD

Natasha Afonso, MD, MPH

Ankita Agarwal, MD

Salman Ahmad, MD, FACS, FCCM, MS

Sumera Ahmad, MD

Mubbasheer Ahmed, MD

Leanne Aitken RN, PhD

Kwame Akuamoah-Boateng, DNP, ACNP-BC, FCCM

Daniela Alampi, MD

Sheila Alexander, PhD, RN

Peta Alexander, MBBS, FRACP, FCICM

Kaitlin Alexander, PharmD

André Alexandre, MD

Ayham Alkhachroum, MD

Theodore Alston, MD, PhD

Javier Amador-Castaneda, BHS

Shashikanth Ambati, MBBS

Pouya Ameli, MD, MS

Sarah Andersen, MD, MS

Lisa Anderson-Shaw, DrPH, MA, MSN, HEC-C

George Anesi, MD, MSCE, MBE

Umair Ansari, PharmD, MBA

Andrew Argent, MBBCh, MD

Donna Armaignac, PhD, APRN, CCNS, CCRN-K

Janhavi Athale, MD

Trina Augustin, MD

Georg Auzinger, MD, AFICM, EDIC, FRCP

Razvan Azamfirei, MD, PhD

James Bailey, MD, PhD

Jonathan Ball, MSc, MD, FCICM, FFICM, FRCP, EDIC,

Ramani Balu, MD, PhD

Oyshik Banerjee, PharmD

Kaysie Banton

Carmen Barbas, MD, PhD

Philip Barie, MD, FCCM

Dalia Bashir, MD, MBBS

Gary Bass, MD, MBA, PhD, MRCS, MRCSI, FEBS

Andriy Batchinsky, MD

Seth Bauer, PharmD, FCCM

Philippe Bauer, MD, PhD, FCCM

Kevin Baumgartner, MD

Rachel Beekman, MD

John Berkenbosch, MD

Pavan Bhatraju, MD

Luca Bigatello, MD

Leanne Boehm, PhD, RN, ACNS-BC, FCCM

Santiago Borasino, MD, MPh

Mehmet Bosnak, MD

Andrew Boyle, PhD, MBChB

David Bracco, MD, PhD, FCCM, FRCS(C)

Ricardo Branco, PhD

Richard Branson, MSc, RRT

Sue Brierley-Hobson MSc

Meredith Broberg, MD

Nicolas Bruder, MD

John Bruno, MD

Samuel Brusca, MD

Maria Bruzzone, MD

Marilyn Bulloch, PharmD, BCPS

Brandi Buscemi A.S.

Warwick Butt, MD

Mark Caridi-Scheible, MD

Ana Carlotti, MD

Thiago Carneiro, MD, MPH

David Carpenter, MPAS

Anne-Marie Carpenter, MD

Lane Carrandi

Fernanda Carvalho Poyraz, MD, PhD

Beulah Castor, MD

Kelly Cawcutt, MD, MS

Kelly Cawcutt, MD, MS

Maurizio Cereda, MD

Donald Chalfin, MD, MS, MPH, FCCM

Arjun Chatterjee, MD, MS

Jen-Ting Chen, MD, MS

Linda Chlan, PhD, RN, FAAN

Benjamin Chousterman, MD, PhD

Emuejevoke Chuba, MD, MSc

Thomas Ciesielski, MD

Amalia Cochran, MD

Michael Connor Jr., MD

Andrew Conway Morris, MB ChB, MRCP, PhD, FRCA, FFICM

David Cook, PhD, FCICM, FANZCA, MB BS

Craig Coopersmith, MD

Claire Creutzfeldt, MD

Matthew Cummings, MD

Ivan da Silva, MD, PhD

Rita D’Aoust, PhD, ACNP, ANP-BC

Auguste Dargent, MD, PhD

Michael Darmon, MD, PhD

Sagar Dave, DO

Kali Dayton, DNP

Akash Deep, MD, FRCPCH

Samuel Demaria Jr

John Devlin, PharmD, FCCM

Monica Diaz, MD

Caoimhe Duffy, MD, MSc

Siddharth Dugar, MD

Melody Duvall

Adam Dziorny, MD, PhD

Philip Efron, MD

Wasim El Nekidy, PharmD

Heidi Engel, PT, DPT

Perenlei Enkhbaatar, MD, PhD

Jose Eduardo Espindola Lima, MD

Laura Evans, MD, MSc

Evan Ewers, MD, MPH

James Fackler, MD, FCCM

Ericka Fink, MD, MS

David Fischer, MD

Babar Fiza, MD

Brandon Foreman, MD MS

Rafael Fricks, PhD

Jennifer Frontera, MD

Susan Gaeta, MD

Bhavita Gaglani, MD

Hannah Galvan, MPAS, DMSc

Jonathan Garcia Ramiu, MD

Nicolas Gaspard, MD, PhD

Garry Gellert, DPT

Anthony Gerlach, PharmD, BCPS, FCCM

Mikhael Giabicani, MD

Jasreen Gill, MBBS, CCRP

Shweta Goswami, MD

Loreta Grecu, MD

Colin K. Grissom, MD

Neil Halpern, MD, MCCM

Gavin Harris, MD

David Harrison, PhD

Joanna Hart, MD

Ilana Harwayne-Gidansky, MD, MA, FAAP, CHSE

Hesham Hassaballa, MD

Daithi Heffernan, MD, MBA, FACS, MRCSI

Vitaly Herasevich, MD, PhD, MSc

Aaron Mark Hernandez, MD

Archana Hinduja, MD

Maxwell Hockstein, MD, MS

Carol Hodgson, PhD, PT, FACP, MPhil

Andre Holder, MD, MSc

Steven Hollenberg, MD, FCCM

Mark Hoofnagle, MD, FACS

Christopher Horvat, MD, MHA

Richard Hotchkiss, MD

Steven Hsu, MD

Kendall Huntt, PharmD

Robert Hyzy, MD

Craig Jabaley, MD

Judith Jacobi, PharmD, MCCM, BCCCP

Matthew Jaffa, DO

Mary Jarzebowski, MD

Archana Jayaraman, PhD

Marc Jeschke, MD, PhD

Asumthia Jeyapalan, DO, MHA

Shixie Jiang, MD

Gideon Johnson, PhD, RN

Rishikesan Kamaleswaran, PhD

Prem Kandiah, MD

Sandra Kane-Gill, PharmD, MSc, FCCM, FCCP

Peter Kanizsai, MD, PhD

Lewis Kaplan, MD, FACS, FCCP, FCCM

Tarun Kapoor, MD

Kunal Karamchandani, MD

Vern Kerchberger, MD

Jozef Kesecioglu, MD, PhD

Imad Khan, MD

Ashish Khanna, MD, FCCP, FCCM, FASA

Michelle Kho, PT, PhD

Kevin Kilgallon, MD

Jennifer Kim, MD, PhD

Tyree Kiser, PharmD

Dana Klavansky, MD

Ruth Kleinpell, PhD, RN

Nathan Klingensmith, MD

Matthew Koch, MD

Lauren Koffman, DO, MS

Marin Kollef, MD

Teresa Kortz, MD, MS, PhD

Karen Korzick, MD MA

Natalie Kreitzer, MD, MS

Erin Kritz, DO

Yi-Chen Lai, MD

Fong Lam, MD

Giovanni Landoni, MD

Michael Lanspa, MD, MS

Liza Laquian, MD

Javier Lasa, MD

Jan Hau Lee, MBBS, MRCPCH, MCI

Laurie Lee, MN, PhD

Matthieu Legrand, MD, PhD

Stephane Legriel, MD, PhD

Marc Leone, MD, PhD

Jerrold Levy, MD

Leo Liu, MD

Tyler Loftus, MD

Micah Long, MD

András Lovas, MD, EDAIC

Brandon Lucke-Wold, MD, PhD

Patrick Lyons, MD

Robert MacLaren, PharmD

Kate Madden, MD, MMSc

Jihad Mallat, MD, PhD

Halinder Mangat, MD, MSc

Robert Mankowski, PhD

Niels Martin, MD

Patricia Martinez-Quinones, MD, PhD

Ryan Maves, MD

Anthony McGuire, PhD, ACNP-BC, FAHA

Bairbre McNicholas, MB BCh, FRCPI, PhD, FJFICMI

Heather Meissen, DNP, ACNP

Andrew Miller, MSc

Danielle Miltz, PA-C

Lyle Moldawer, PhD

Kelli Money, MD, PhD

Peter Moran, PharmD

Jarrod Mosier, MD

Susanne Muehlschlegel, MD, MPH

Ali Naqvi, MD

Boulos Nassar, MD

Lama Nazer, PharmD

Andrea Nei, PharmD

Axel Nierhaus, MD, EDIC

Ng Niu, MSN

Vanessa Nomellini, MD, PhD

Mark Nunnally, MD

Peter Nydahl, PD, PhD

Paul Nyquist, MD

Frank O’Connell, MD

Uchenna Ofoma, MD, MS

Vanessa Okolie, DNP

Segun Olusanya, MD

Tarig Omer, MD

John Oropello, MD, MCCM

Lars Palmowski, MD

Jeremy Pamplin, MD, FCCM, FACP

Margaret Parker, MD, MCCM

Stephen Pastores, MD, FCCM

Ronald Pauldine, MD

Ithan Peltan, MD, MSc

Silvia Perez-Protto, MD

Michael Pienaar, PhD

Jefferson Piva

Michael Pizzi, DO, PhD

David Porembka DO, FCCM

Hallie Prescott, MD, MSc

Richard Pugh, MSc, MB ChB

Tim Rahmel, MD

Rupesh Raina, MD

Swarna Rajagopalan, MD, MS

Venkatakrishna Rajajee, MD

Hariprem Rajasekhar, MD

Pam Ramsay, PhD, BSc, RN

James Reynolds, PhD

Chanu Rhee, MD, MPH

Michael Ripple, MD, PhD

Russel Roberts, PharmD, FCCM, BCCCP

Angela Rogers, MD, MPH

Michael Ruppe, MD

Katerina Rusinova, MD

Derek Russell, MD

Ahmed Said, MD, PhD

Lina Saliba, PharmD

L. Sanchez-Pinto, MD, MBI

Gaetano Santulli, MD

Aarti Sarwal, MD, FCCM, FNCS, FAAN, RPNI

Richard Savel, MD

Emily Sayilgan, ACNP-BC

Dane Scantling DO, MPH

Sara Scarlet, MD, MPH

Benjamin Scott, MD

Chail Shah, MD

Neel Shah, MD

Vishank Shah, MD

Dariush Shahsavari, MD

Asha Shenoi, MD

Bethany Shoulders, PharmD, BCCCP

Lori Shutter, MD

Jon Silversides, PhD

Steven Simpson, MD

Nicole Siparsky, MD, FACS, FCCM

Charlie Slowly, MB, BCh, BAO

Anthony Sochet, MD, MSc

Isaac Solomon, MD, PhD

Michael Spaeder, MD

Zoltan Spolarics, MD, PhD

Roshni Sreedharan, MD, FASA, FCCM

Anne Stey, MD, MSc

Jeffrey Strich, MD

Thomas Strom, PhD, MD

Daniel Sweeney, MD

Sandy Swoboda RN

Ahmed Taha, MD

Maximiliano Tamae Kakazu, MD

Robert Tasker, MD, MBBS

Ken Tegtmeyer, MD

Ravi Thiagarajan, MBBS, MPH

Samuel Tisherman, MD, FCCM

Adalberto Torres, MD, MS, FCCM

Quincy Tran, MD, PhD

Sebastian Tume

H. Michael Ushay, MD, PhD

Asad Usman, MD, MPH

Jean-Louis Vincent, MD, PhD, FCCM

Nicolas Weiss

Douglas White, MD, MAS

Patrick Wieruszewski, PharmD, FCCM

Whitman Wiggans, MD

Adrian Wong, MD

An-Kwok Wong, MD, PhD

Arno Zaritsky, MD

Jerry Zimmerman, MD, PhD, MCCM

Lingyun Zou, PhD

